# Current Landscape of NRF2 Biomarkers in Clinical Trials

**DOI:** 10.3390/antiox9080716

**Published:** 2020-08-07

**Authors:** Yoko Yagishita, Tonibelle N. Gatbonton-Schwager, Melissa L. McCallum, Thomas W. Kensler

**Affiliations:** Fred Hutchinson Cancer Research Center, Translational Research Program, Public Health Sciences Division, Seattle, WA 98109, USA; yyagishi@fredhutch.org (Y.Y.); tgatsch@fredhutch.org (T.N.G.-S.); melissalmccallum@gmail.com (M.L.M.)

**Keywords:** biomarkers, NRF2 (nuclear factor erythroid 2 related factor 2), sulforaphane, oltipraz, bardoxolone methyl, dimethyl fumarate, oxidative stress, inflammation, gene expression, carcinogenesis

## Abstract

The transcription factor NF-E2 p45-related factor 2 (NRF2; encoded by *NFE2L2*) plays a critical role in the maintenance of cellular redox and metabolic homeostasis, as well as the regulation of inflammation and cellular detoxication pathways. The contribution of the NRF2 pathway to organismal homeostasis is seen in many studies using cell lines and animal models, raising intense attention towards targeting its clinical promise. Over the last three decades, an expanding number of clinical studies have examined NRF2 inducers targeting an ever-widening range of diseases. Full understanding of the pharmacokinetic and pharmacodynamic properties of drug candidates rely partly on the identification, validation, and use of biomarkers to optimize clinical applications. This review focuses on results from clinical trials with four agents known to target NRF2 signaling in preclinical studies (dimethyl fumarate, bardoxolone methyl, oltipraz, and sulforaphane), and evaluates the successes and limitations of biomarkers focused on expression of NRF2 target genes and others, inflammation and oxidative stress biomarkers, carcinogen metabolism and adduct biomarkers in unavoidably exposed populations, and targeted and untargeted metabolomics. While no biomarkers excel at defining pharmacodynamic actions in this setting, it is clear that these four lead clinical compounds do touch the NRF2 pathway in humans.

## 1. The KEAP1-NRF2 System

It has been more than 20 years since the initial molecular description of the Keap1-Nrf2 signaling pathway, wherein it was recognized as an adaptive response pathway to exogenous [1], and later as an adaptive response pathway to endogenous stresses [2]. Nuclear factor erythroid 2 related factor 2 (NRF2) is a transcription factor that regulates the expression of over 300 target genes with roles in antioxidant and anti-inflammatory actions, electrophile detoxication, cell metabolism, proliferation and differentiation, and general cytoprotection. Kelch-like ECH-associated protein-1 (KEAP1) is an adaptor protein for the Cullin-3 ubiquitin ligase and a key cytoplasmic repressor of NRF2. KEAP1 interaction with NRF2 leads to NRF2 polyubiquitination and its subsequent proteasomal degradation. In the presence of oxidative stress or inducers, which are often electrophiles, key “sensor” cysteine thiol groups on KEAP1 are modified, disrupting the degradation process and allowing for nascent NRF2 to directly translocate into the nucleus and to target gene transcription. Target genes are defined by the presence of functional antioxidant response elements (AREs) in their regulatory regions. NRF2 heterodimerizes with one of the small musculo-aponeurotic fibrosarcomas (sMAF) to bind to the ARE. Sentinel downstream targets include NAD(P)H: quinone oxidoreductase 1 (*NQO1*), heme oxygenase 1 (*HMOX1*), glutamate-cysteine ligase catalytic subunit (*GCLC*), and the glutathione-S-transferases (*GSTs*). The rapid inducibility of a response based on a de-repression mechanism is an important feature of this cytoprotective KEAP1-NRF2 system. An additional feature is the extensive crosstalk between NRF2 and other transcription factor signaling pathways, allowing for further fine-tuning of physiological responses to stress. There are excellent reviews that document the historic milestones in the characterization of the pathway, the molecular mechanisms governing the functions of KEAP1 and NRF2, as well as their roles in physiology and pathology [3,4,5,6].

## 2. Pharmacological Inducers of KEAP1-NRF2 Signaling

A plethora of preclinical studies, utilizing cell culture models as well as animals, continues to lead to the discovery and characterization of molecules that could induce (or inhibit) the pathway and mitigate toxicities or diseases driven by exposures to electrophiles, oxidants, and intrinsic inflammatory processes. The profound protective actions of many classes of “Nrf2 inducers” in rodent models of acute and chronic diseases, as well as corresponding actions in genetically manipulated models (ranging from *C. elegans, Drosphila*, and zebrafish to mice and rats) in which elements of the pathway were disrupted or amplified, led to the notion that NRF2 signaling in humans might be a propitious target for disease prevention and treatment [5,7,8]. As reviewed elsewhere [9,10,11,12], many drugs and natural products, exhibiting remarkable structural diversity, are under development and clinical evaluation for these purposes. One of many features retarding drug development is the lack of sensitive and specific biomarkers that can rapidly inform understandings of dose-responsiveness, mechanistic specificity, as well as magnitude and duration of pharmacodynamic action in clinical trial settings. Such needs pervade the process of clinical development of NRF2 inducers. Although hundreds—if not thousands—of molecules have been described as inducers and dozens are in clinical development, only four (dimethyl fumarate (DMF), bardoxolone methyl (BARD-Me), oltipraz, and sulforaphane (SFN)) have appeared substantively in the peer-reviewed literature, especially wherein measures of biomarkers have been a component of the clinical protocols. Interestingly, all four of these agents intersect the NRF2 signaling pathway through interactions with cysteine151 in KEAP1 [5]. While this review focuses on biomarkers likely related to NRF2 action, because of their reactivity with thiols all these agents affect multiple targets and pathways in cells. Sole attribution of any response to actions on NRF2 in the context of the biomarkers reported herein has not been firmly established. Nonetheless, there are common themes in the field, and as mechanistic insights become sharpened along with analytic improvements higher confidence in the specificity and sensitivity of some of these biomarkers may evolve.

**Figure 1:**
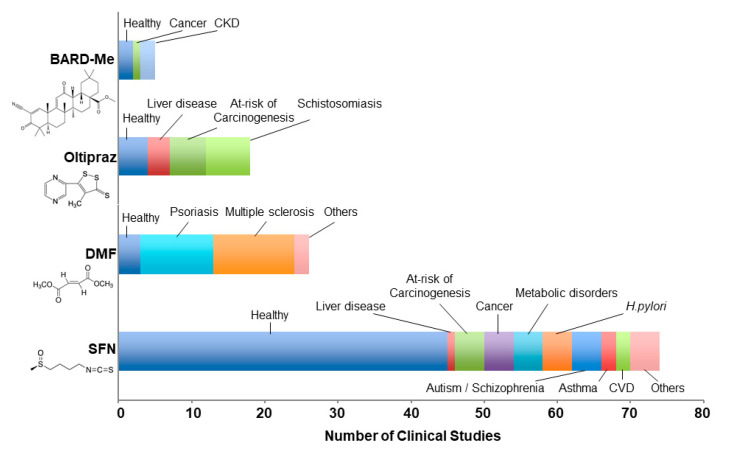
Study populations examined in peer-reviewed clinical studies of nuclear factor erythroid 2 related factor 2 (NRF2) inducers. Studies for bardoxolone methyl (BARD-Me), oltipraz, dimethyl fumarate (DMF), and sulforaphane (SFN) totaled 5, 18, 26, and 75, respectively, from 1982 to June 2020. Literature searches were conducted on PubMed, Google Scholar, and ClinicalTrials.gov. Publications based on the same clinical trial were aggregated as one study in this graph. The chemical structure for each NRF2 inducer is indicated below each name. CKD, chronic kidney disease; CVD, cardiovascular disease.

Summaries of the clinical development and utilization of DMF, BARD-Me, oltipraz, and SFN are provided in the following sections, along with highlights of general evidence for the targeting of NRF2 by these agents based on preclinical studies. Interestingly, the clinical applications of each drug are quite distinct. The distributions of healthy and disease-state study populations enrolled in clinical trials with these four agents are highlighted in Figure 1.

### 2.1. Dimethyl Fumarate (DMF, BG-12, Tecfidera)

Fumaric acid esters are used for the treatment of psoriasis and multiple sclerosis. A mixture of dimethyl fumarate with mono ethylfumarate salts was developed as a drug product (Fumaderm^®^) and registered in 1994 as an oral agent for the treatment of moderate to severe plaque psoriasis. Dimethyl fumarate alone (Skilarence^®^) also has EU approval for this indication [13]. Due to the immunomodulatory potential of Fumaderm^®^, DMF was evaluated in other immune-mediated diseases, which led to testing of DMF in large multicenter phase 2 and 3 trials of relapsing–remitting multiple sclerosis [14]. Oral DMF as a galenical formulation (BG-12, Tecfidera^®^) was subsequently demonstrated to be an effective agent [15,16] and was approved for use in this disease by the U.S. Food and Drug Administration in 2013 and the European Medicines Agency in 2014 [17]. New analogs featuring better bioavailability and efficacy are under development [10]. DMF has been linked to several treatment-limiting adverse events, including flushing and gastrointestinal complaints, and less frequently to persistent lymphopenia. Such action likely precludes the use of DMF in any disease prevention settings. A summary of clinical trials utilizing fumaric acid esters, all of which include DMF, is listed in Table S1 [15,16,18,19,20,21,22,23,24,25,26,27,28,29,30,31,32,33,34,35,36,37,38,39,40,41,42,43,44,45,46,47,48,49,50,51,52,53,54,55,56].

DMF is almost completely converted to monomethyl fumarate (MMF) by intestinal esterases. Thus, it is likely that DMF serves as a prodrug in the elaboration of its therapeutic actions, with MMF the likely active form. Talalay reported in 1990 [57] that DMF and other fumaric acid esters induced Nrf2 target gene (*Nqo1* and *Gst*) activities in various organs of mice and rats after dietary administration. DMF and MMF contain double bonds, allowing them to act as Michael acceptors to form adducts with thiol groups, such as C151 in Keap1. Much more recently, Linker et al. [58] demonstrated in mice that oral DMF induces the expression of Nrf2 target genes in multiple cell types of the central nervous system. Studies in Nrf2-disrupted mice indicate a loss of protective effect of DMF in a multiple sclerosis model of experimental autoimmune encephalitis and MPTP-induced experimental Parkinson’s disease [58,59,60]. However, Nrf2-independent actions are also being described. For example, DMF and MMF activate the nicotinic receptor hydroxycarboxylic acid receptor 2, which is expressed in immune cells and gut epithelial cells, resulting in NRF2-independent anti-inflammatory responses, in addition to anti-inflammatory actions associated with Nrf2 signaling. There is substantial evidence that DMF and MMF influence multiple aspects of the immune system that contribute to both the therapeutic effects and its major side effects [10,17].

### 2.2. Bardoxolone-methyl (BARD-Me; CDDO-Me)

Many dozens of synthetic oleanane triterpenoids have been synthesized, guided by efforts to enhance the weak anti-inflammatory activity of the naturally occurring triterpenoid oleanolic acid [61], which in vitro have been shown to: (1) suppress inflammation-like responses and oxidative stress, and therefore to be cytoprotective, especially at low nanomolar doses; (2) induce differentiation; and (3) block cell proliferation and induce apoptosis at higher micromolar doses [62]. Extensive animal data have been described demonstrating efficacy of triterpenoids in the prevention or amelioration of neurodegenerative diseases and in diseases of the eye, lung, cardiovascular system, liver, gastrointestinal tract, and kidney, as well as in cancer and in metabolic and inflammatory or autoimmune disorders [62,63]. Two triterpenoids have been studied extensively in preclinical studies (CDDO-Im; 1-[2-cyano-3-,12-dioxooleana-1,9(11)-dien-28-oyl]imidazole); (CDDO-Me; bardoxolone methyl; methyl 2-cyano-3,12-dioxooleana-1,9(11)dien-28-oate) and are likely to have strongly overlapping, but not entirely concordant, mechanisms of action. As summarized in Table S1, CDDO-Me (BARD-Me) was the featured synthetic oleanane triterpenoid selected by Reata Pharmaceuticals for clinical development [64,65,66,67,68,69,70,71,72,73,74,75].

Over 30 clinical trials with BARD-Me have been registered in ClinicalTrials.gov. The first phase I clinical trial of BARD-Me was conducted in patients with advanced solid tumor and lymphoma to determine the dose-limiting toxicity and the maximum tolerated dose [66]. In this first trial, an increase in estimated glomerular filtration rate (eGFR) was also noted. This observation led to the evaluation of BARD-Me for treatment of patients with chronic kidney disease (CKD) and prompted several follow-up studies, including a phase 2 Bardoxolone Methyl Treatment: Renal Function in CKD/Type 2 Diabetes (BEAM); and phase 3 Bardoxolone Methyl Evaluation in Patients with Chronic Kidney Disease and Type 2 Diabetes Mellitus: the Occurrence of Renal Events (BEACON) trials. While BEAM showed a promising increase in estimated glomerular filtration rate (eGFR), BEACON was terminated early due to heart failure events within the first 4 weeks of treatment [64,67]. Bardoxolone-methyl is currently in clinical trials for Alport’s syndrome (A Phase 2/3 Trial of the Efficacy and Safety of Bardoxolone Methyl in Patients With Alport Syndrome-CARDINAL, NCT03019185), IgA nephropathy, type 1 diabetic nephropathy, focal segmental glomerulosclerosis, and autosomal dominant polycystic kidney disease (A Phase 2 Trial of the Safety and Efficacy of Bardoxolone Methyl in Patients With Rare Chronic Kidney Diseases - PHOENIX, NCT03366337). An additional phase 3 study in patients with diabetic kidney disease is also being performed with a primary outcome of time to onset of a 30% decline in eGFR or end-stage renal disease (A phase 3 study of bardoxolone methyl in patients with diabetic kidney disease, AYAME, NCT03550443) [76].

The role of NRF2 is inferred to be a central component of the mechanism of action of the bardoxolone methyl. CDDO-Im [77] and CDDO-Me [78] protect against acute kidney injury in mice, while Nrf2 null mice are sensitized to injury [79]. Similarly, CDDO-Im completely abrogates aflatoxin hepatocarcinogenesis in rats [80], whilst Nrf2 knockout rats show markedly enhanced sensitivity to the hepatotoxic effects of aflatoxin [81]. Interestingly, although CDDO-Im, CDDO-Me, and DMF all activate the Nrf2 pathway, they target distinct genes and signaling pathways, and in the case of DMF result in opposite effects for the prevention of experimental lung cancer in mice [82]. Highlighting the importance of additional mechanisms of action, Ball et al. [83] recently reported that CDDO-Me relieves immunosuppression in the breast tumor microenvironment and unleashes host adaptive antitumor immunity. This may be mediated in part by conversion of breast-tumor-activated macrophages from a tumor-promoting to a tumor-inhibiting activation state [84]. Some anti-inflammatory actions of CDDO-Me are likely mediated through additional actions on nuclear factor-κB (NF-κB) [85].

### 2.3. Oltipraz

Oltipraz, [4-methyl-5-(2-pyrazinyl)-3H-1,2-dithiole-3-thione], was originally developed by Rhône-Poulenc (subsequently acquired by Sanofi) as a possible treatment for schistosomiasis and was extensively evaluated in clinical trials in the early 1980s. Field trials in Mali, Gabon, Sudan, and other sites, using short courses with durations of 1–5 days with total doses of 1.25–7.5 g, achieved cure rates of greater than 90% [86,87,88,89,90,91]. However, side effects occurred in about 10% of participants, principally related to the digestive system and to fingertip pain, with the latter being amplified by exposure to sunlight. Although all effects were reported as mild, subsided within a few days, and did not require discontinuation of the drug, the concerns regarding photosensitivity led to the abandonment of oltipraz for the treatment of schistosomiasis. This decision was surely influenced by concurrent clinical progress of less expensive, equi-effective, and less problematic drugs for the chemotherapy of schistosomiasis.

While studying mechanisms of antischistosomiasis by oltipraz, Ernest Bueding and colleagues at Johns Hopkins initially noted that giving the drug to mice infected with *Schistosoma mansoni* caused a dramatic reduction in the glutathione stores of the parasite, while paradoxically markedly elevating glutathione levels in many tissues of the host [92]. Subsequent studies demonstrated that oltipraz and some structurally related 1,2-dithiole-3- thiones were potent inducers of enzymes concerned with the maintenance of reduced glutathione pools, as well as enzymes important to electrophile detoxication in multiple tissues of rats and mice [93]. These results prompted Bueding to predict that oltipraz might have cancer chemopreventive properties [94], and that perhaps at lower doses the side effects would be mitigated.

Largely under the aegis of the drug development program of the Chemoprevention Branch of the National Cancer Institute, oltipraz underwent extensive evaluation for anticarcinogenic efficacy in animal models. As reviewed elsewhere [95,96], oltipraz has shown chemopreventive activity against different classes of carcinogens targeting the trachea, lung, stomach, small intestine, colon, pancreas, liver, urinary bladder, mammary gland, hematopoietic cells, and skin. The most dramatic actions of oltipraz occurred in the colon and liver, where dietary administration resulted in significant reductions in both tumor incidence and multiplicity. Accordingly, subsequent clinical trials of oltipraz (Figure 1; Table S1 [86,87,88,89,90,91,97,98,99,100,101,102,103,104,105,106,107,108,109,110,111] focused on cancer-preventive interventions targeted to the colon and liver, and most recently mitigation of hepatic fibrosis and nonalcoholic fatty liver disease (NAFLD). Only one current trial with oltipraz (NCT04142749: A Multi-center, Randomized, Double-blind, Placebo-controlled, Parallel, Phase II Clinical Trial to Evaluate the Efficacy and Safety of Oltipraz) is listed on ClinicalTrials.gov, indicating that while this agent has provided historical perspectives on biomarker utilization directed towards the NRF2 pathway, it appears to be at a defining point for a possible clinical path forward.

A role for the involvement of Nrf2 in the chemopreventive actions of oltipraz in animals was built on the foundation of induction of Nrf2 target genes and enzymes in multiple rodent tissues [93], amplified by an appreciation for the role of AREs in mediating these responses [112] and capped by studies demonstrating that protective effects of oltipraz against DNA adduct formation and tumorigenesis were abrogated in Nrf2-disrupted mice [113,114]. These observations, in turn, guided the choice of biomarkers selected as intermediate endpoints for clinical studies with oltipraz.

### 2.4. Sulforaphane (SFN)

Sulforaphane was described in the middle of the last century as an antibiotic, and was isolated from red cabbage and from the western USA rangeland weed hoary cress. Various groups have since synthesized it, however Talalay and Zhang were the first to isolate it from broccoli [115] and to demonstrate its cancer protective properties (against mammary carcinogenesis in rats) [116]. Its biogenic precursor, glucoraphanin, was then found in abundance in broccoli sprouts. Sprout-based preparations were confirmed to be active for prevention in animal carcinogenesis models [117].

The highly reactive isothiocyanate SFN is produced in plants as an inert precursor, the glucosinolate glucoraphanin. Upon disruption of plant tissue integrity, glucoraphanin interacts with myrosinase, which catalyzes the hydrolysis of glucoraphanin to yield SFN. The β-thioglucosidases within the human microbiome also catalyze this bioactivation. Glucoraphanin occurs in all tissues of broccoli plants, though it is most abundant in the aerial portions, and the developing florets and ultimately the seeds are richest in this compound [117]. Studies to examine the pharmacokinetics of SFN in humans began in 1998 [118]. In the subsequent 75 or so studies examining pharmacokinetics, pharmacodynamics, or efficacy, a multitude of formulations have been used. Broccoli-based preparations have consisted typically of either glucoraphanin; SFN; glucoraphanin with added active myrosinase; the raw, cooked, or dried vegetables themselves (either broccoli or broccoli sprouts); or extracts of broccoli seeds or sprouts, which are glucoraphanin-rich, SFN-rich, or both. This diversity of formulations—and thus lack of consistency—has provided uncertainty regarding actual administered doses and confusion in the interpretation of study results. The broccoli (sulforaphane) literature with regards to formulation, dose, and response in preclinical and clinical studies has been reviewed recently [11]. Relatively few studies have evaluated the efficacy of SFN in its various formulations against clinical endpoints of disease. As depicted in Figure 1, more than half of the published studies have involved healthy volunteers. However, in part based on a broad spectrum of beneficial responses observed in animal models, clinical trials focused on neurodevelopmental diseases such as autism and schizophrenia, cardiovascular disease, sickle cell anemia, chronic obstructive pulmonary disease, asthma, *Helicobacter pylori* infection, and environmental factors contributing to carcinogenesis, as well as diabetes, metabolic syndrome, and related disorders have been undertaken (reviewed in [11,119] and are listed in Table S1 [118,120,121,122,123,124,125,126,127,128,129,130,131,132,133,134,135,136,137,138,139,140,141,142,143,144,145,146,147,148,149,150,151,152,153,154,155,156,157,158,159,160,161,162,163,164,165,166,167,168,169,170,171,172,173,174,175,176,177,178,179,180,181,182,183,184,185,186,187,188,189,190,191,192,193,194,195,196]).

Bioassay guided fractionation of acetonitrile extracts of SAGA broccoli led to the isolation of SFN as the major inducer [115]. The bioassay tracked the induction of Nqo1 activity in murine hepatoma cells. Thus, a Nrf2 target gene was at the center of its isolation from broccoli. There are now over 300 publications in which the actions of SFN in mice invoked a role for Nrf2. Studies in which these actions are diminished or abrogated in parallel experiments in Nrf2-disrupted mice provide the strongest lines of evidence for a key role of this transcription factor in its actions. With that said, it is equally evident that other modes of action contribute to the molecular responses to SFN in animals and humans [119,197,198]. Such polypharmacy may well contribute to the efficacy of the agent in disease prevention and mitigation but obfuscates the value of specific pharmacodynamic biomarkers in the clinical development and evaluation of SFN—perhaps even more so, because unlike the situation with triterpenoids (e.g., CDDO-Im and CDDO-Me), where there appears to be a hierarchical activation of targets/pathways with increasing dose [62], it is more likely that the multiple targets of SFN are activated at similar concentrations [198].

### 2.5. Other Natural Product Inducers

While SFN is generally considered to be the most potent natural product inducer of Nrf2 signaling, there are many plant-derived molecules that activate the pathway. They have been identified principally in cell culture screens. Several comprehensive reviews of this topic have been published recently [193,199]. These molecules can be natural products, natural product-derived, or natural product-inspired. Moreover, they can activate the pathway through different mechanisms—almost all intersecting with Keap1 directly or indirectly. However, relatively few have seen rigorous evaluation in clinical studies or trials. Some natural products of clinical use include curcumin, resveratrol, and flaxseed.

The polyphenol curcumin, which is isolated from *Curcuma longa* and provides yellow color to the spice turmeric, is a weak inducer of Nrf2 signaling in cell culture [200]. Long used in Ayurveda for the treatment of many conditions, two clinical trials of curcumin have examined NRF2-driven biomarkers. Yang et al. [201] reported that curcumin (500 mg, daily) induced *NQO1* levels in lymphocytes and reduced plasma malondialdehyde levels in plasma of T2DM patients. Modulation of inflammatory biomarkers was also observed. Jiménez-Osorio et al. [202] failed to observe any effects of curcumin (320 mg/d for 8 weeks) on the activities of NRF2 target genes (glutathione peroxidase, glutathione reductase, superoxide dismutase (*SOD*), and catalase) in peripheral blood mononuclear cells (PBMCs) of diabetic proteinuric chronic kidney disease (CKD) patients enrolled in a placebo-controlled, double-blind trial. Similar mixed results were seen in two placebo-controlled, randomized trials with resveratrol, another polyphenol, which in this instance is found in the skins of grapes and berries and is widely marketed as a dietary supplement. Saldanha [203] reported no effect of 500 mg/d on NRF2 expression in peripheral blood mononuclear cells (PBMCs) of non-dialized CKD patients, whilst Seyyedbrahimi et al. [204] observed significant changes in *NRF2* and *SOD* levels with 400 mg twice-daily in T2DM patients. Flaxseed, a dietary botanical supplement with high fiber, lignan phenolics, and omega-3 fatty acids, has anti-inflammatory and antioxidant properties in murine models of acute and chronic lung injury. Ten cystic fibrosis patients and five healthy volunteers consumed 40 g of flaxseed daily for 4 weeks in a pilot study of tolerability and possible pharmacodynamic action. No significant effects were observed on biomarkers of NRF2 signaling or attenuation of oxidative stress. Thus, the translation of these broadly touted natural products towards effective modulation of NRF2 signaling and possible disease mitigation remain largely unfulfilled. This outcome likely reflects the relative lack of potency of these molecules and the myriad of molecular targets that may be modified.

## 3. Biomarker-Based Clinical Studies and NRF2 Inducers

Prospective clinical studies have revolutionized the development of medicine by providing reliable evidence on the efficacy and safety of novel treatment strategies. In newer paradigms of drug development, biomarkers are often used to guide early clinical development (e.g., phase 0 studies). A biomarker is defined as “a characteristic that is objectively measured and evaluated as an indicator of normal biological processes, pathogenic processes, or pharmacologic responses to a therapeutic intervention” [205]. A simple distinction can be made between clinical biomarkers and mechanism-specific biomarkers. Clinical biomarkers are thought to reflect disease activity and pathophysiology, which are not discussed in any detail in this article. The mechanism-specific biomarkers, which are the main focus of this article, reflect the molecular action of an agent on the pharmacology or pathophysiology, where biomarkers are used to evaluate pharmacokinetic and pharmacodynamic aspects, including target activation [206].

**Figure 2:**
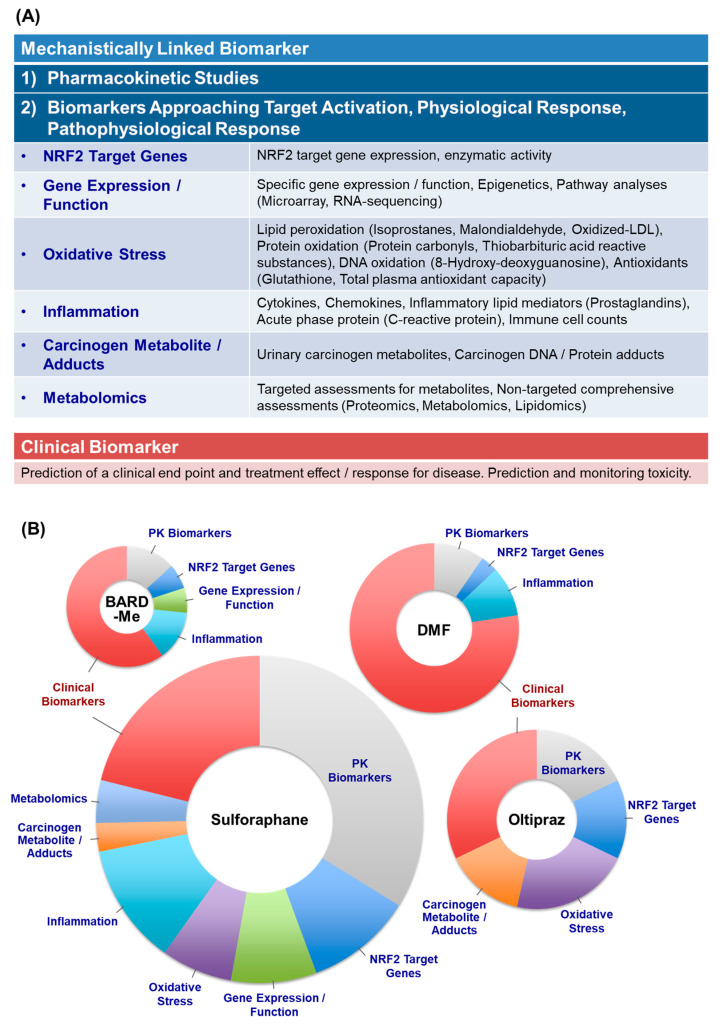
(**A**) Classification of biomarkers used in published clinical studies of “NRF2 Inducers”. (**B**) Distribution of biomarker categories used in the clinical studies. The size of each pie reflects the total number of times a biomarker of each class was used in the clinical studies. Multiple classes of biomarkers were used in some studies. PK, pharmacokinetics.

Screening of peer-reviewed clinical studies of NRF2 inducers from 1982 to June 2020 (Table S1) identified 12, 21, 41, and 78 published studies for BARD-Me, oltipraz, DMF, and SFN, respectively. Not all studies utilized mechanistic biomarkers. The most frequently used mechanistic biomarkers are enumerated in Figure 2A, according to their placement in 6 broad categories, which are reflective of NRF2 and other modes of action. To give some context regarding the actual usage of these biomarkers, the distributions of these biomarkers in the clinical studies with each of the 4 agents were further analyzed (Figure 2B). There is a varied landscape with unique distributions for each clinical agent. As the NRF2 signaling pathway is a new and evolving proposed molecular target for clinical application, mechanistically linked assessments within clinical studies would a priori seem to be fundamental. Indeed, several mechanically linked biomarkers have been investigated widely in the clinical studies for SFN and oltipraz, agents that were developed largely in academic settings. Presumably, these biomarkers have been used to guide selection of dose or formulation in advance of efficacy trials. On the other hand, within the clinical studies for BARD-Me and DMF, agents developed by Pharma, clinical biomarkers account for the majority usage in publications. Here, mechanistic biomarker use tends to be confirmatory once some efficacy has been established.

Although standardizing NRF2 biomarker definitions presents a challenge as there is a significant overlap in biomarker categories, here we breakdown and describe each mechanistically linked biomarker that can be potentially modulated by NRF2 inducers.

### 3.1. Nrf2 Target Genes

Quickly after its discovery, it was established in mice that Nrf2 regulates the expression of several hundreds of genes, encoding a network of enzymes involved in a broad-based cellular defense system in mice [6,8]. Less extensive cataloging has been undertaken in humans; however, there appears to a largely concordant, albeit likely more limited set of NRF2 target genes [207,208,209]. Thus, tracking changes in the expression or activities of NRF2-targeted enzymes could be one of the most direct biomarkers for monitoring the effects on the downstream pathway of NRF2 signaling. NAD(P)H: quinone oxidoreductase-1 (NQO1) and heme oxygenase-1 (HMOX1) are widely examined NRF2-targeted enzymes exhibiting sensitivity and reliability of their quantification, wherein the mRNA expression levels of these genes have been evaluated in clinical samples, such as in PBMCs and biopsy samples from study subjects.

Reduced glutathione (GSH; the oxidized form of which is glutathione disulfide, GSSG) is the most abundant cellular antioxidant and a major thiol utilized for detoxication of reactive intermediates. NRF2 regulates the expression of enzymes involved in the synthesis and recycling of GSH, such as the catalytic and modulator subunits of glutamate–cysteine ligase (GCLC and GCLM), glutathione reductase (GR), glutathione peroxidase (GPX), and several glutathione S-transferases (GSTs). Moreover, several proteins within the redoxin family, such as thioredoxin (TRX), thioredoxin reductases (TrxRs), peroxiredoxins (Prxs), and sulfiredoxins (SRXNs), are all regulated by NRF2 and provide compartmentalized sensing and signal transduction of regional production of reactive oxygen species. Thus, the redox regulation by NRF2-targeted genes can modulate the level of oxidative stress, which is considered to be one of therapeutic targets by NRF2 inducers. In this article, these genes are considered as NRF2 target gene biomarkers, as distinguished from oxidative stress biomarkers. *GCLC*, *GCLM*, *GPX*, and *GST* have been used as biomarkers for several clinical studies of NRF2 inducers [104,107,134].

### 3.2. Gene Expression and Function

In addition to prototypic NRF2 target genes, other genes are also examined as biomarkers, where links to NRF2 signaling are more tenuous or perhaps nonexistent. Mounting reports describe how SFN affects multiple potential downstream pathways, such as epigenetic alterations and heat shock. Epigenetic effects mediated through modulation of histone deacetylase (HDAC) activity have been explored in several clinical studies and may relate to the chemopreventive effects of SFN [129,135,140,159,170]. In a study targeting autism spectrum disorder, heat shock proteins were employed as biomarkers reflecting therapeutic effects by SFN [195]. Comprehensive, unbiased analyses for gene expression (e.g., microarray, RNA-sequencing, and ChIP-seq) have provided powerful assessments for gene expression profiling and transcriptional networks. Several studies, principally focused on SFN, have been performed [128,131,193], revealing characteristic and novel gene expression profiling correlated with intervention.

Rajendran et al. [170] reported that wild-type mice, which are more susceptible to dimethylhydrazine-induced colon carcinogenesis than Nrf2-deficient mice, had higher HDAC levels globally and locally. SFN treatment reduced tumor burden, most notably in the wild-type mice, and reduced HDAC3 expression. Thus, Nrf2 status may influence HDAC levels and signaling by its downstream targets, including p16.

### 3.3. Oxidative-Stress-Mediated Biomarkers

Oxidative stress has been defined as “an imbalance in pro-oxidants and antioxidants, with associated disruption of redox circuitry and macromolecular damage” [210]. Measuring oxidative stress in the context of clinical trials has a long and somewhat unsuccessful history, especially relating to intervention studies with “traditional” direct antioxidant compounds. In accord with the definition, many oxidative markers found in the body have been proposed, including lipid peroxidation products, such as malondialdehyde (MDA), isoprostane products, oxidized low-density lipoproteins (LDL), hydroperoxides, and 4-hydroxynonenal; protein oxidation products, such as thiobarbituric acid reactive substances (TBARS); carbohydrate oxidation products, such as 3-nitrotyrosine; and nucleic acid oxidation products, such as 8-hydroxy-2-deoxyguanosine (8-OHdG) [211,212]. These biomarkers, based on molecular oxidation products, have also been applied in trials with NRF2 inducers. As alternatives to measures of oxidation products, tests measuring total oxidative status (TOS), total antioxidant capacity (TAC), and oxidative stress index (OSI) have also been used as oxidative stress markers for clinical studies with NRF2 inducers [138,175]. TOS and total antioxidant status (TAS) are usually used to measure the overall oxidation status or antioxidant status of the body, the ratio of which is expressed as the OSI. The TAC measures the amount of free radicals scavenged by a test solution and is used to evaluate the antioxidant capacity of biological samples. GSH and its oxidized form GSSG in blood are global indices of the redox status in the whole organism. In the case of NRF2 inducers, GSH measurements attempt to reflect the balance between oxidant stress and the biosynthesis of GSH, as well as the recycling of oxidized glutathione through genes responding within the NRF2 downstream pathway. More comprehensive means to assess redox dynamics have been developed by monitoring the reversible oxidation of sulfur-containing amino acids and peptides, notably GSH. The redox states of glutathione/glutathione disulfide (GSH/GSSG) and cysteine/cystine (Cys/CySS) are oxidized in association with several known oxidative-stress-related exposures, health conditions, and measures of physiologic function [210]. Applications to NRF2-targeting drugs have not been undertaken to date.

**Figure 3:**
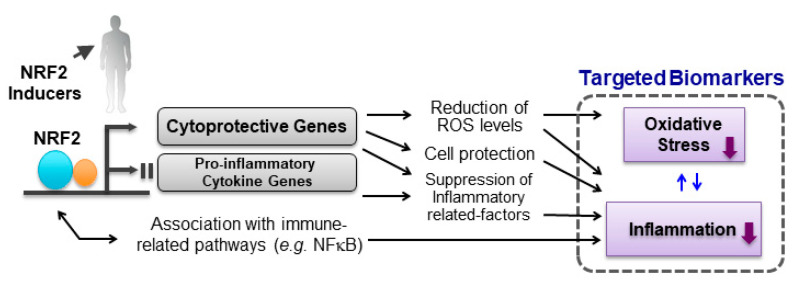
NRF2 signaling pathway and biomarkers targeting oxidative stress and inflammation. ROS, reactive oxygen species.

Nrf2 knockout (*Nrf2*^−/−^) mice are more susceptible to oxidative-stress-based diseases [213]. Many lines of animal experiments show that Nrf2 activation by pharmacological or genetic approaches significantly reduces cellular damage caused by oxidative stress and suppress the development of several kinds of diseases [214,215]. Because an increased oxidative stress is crucial in the pathogenesis of several kinds of diseases in humans, the notion of targeting oxidative stress with NRF2 inducers has been expanding. As mentioned above, the target genes of NRF2 include genes involved in the regulation of the synthesis and conjugation of GSH. Supported by molecular biology and in vivo experimental studies, assessments of changes in levels of oxidative stress biomarkers could be one important means for optimizing clinical studies of NRF2 inducers (Figure 3).

### 3.4. Inflammation-Mediated Biomarkers

The oldest definition of inflammation is by Aulus Cornelius Celsus, who defined the four hallmarks of inflammation: “rubor, et tumor, cum calore, et dolore”, meaning redness, swelling, heat, and pain [216], respectively, which are described as the cardinal signs of inflammation. Surprisingly, the concept of inflammatory biomarkers was established in the first century AD. Studies investigating the molecular basis of inflammation have led to the identification of markers that may also serve as new targets of therapy for inflammation-related diseases. Inflammation and oxidative stress are tightly linked. Although oxidative stress biomarkers mainly approach the formation of effector molecules, such as lipid peroxides and oxidized proteins of DNA, the inflammatory markers measure the response of the organism, for example through the production of inflammatory cytokines and lipid mediators (Figure 2A) [217].

Emerging evidence demonstrates the linkage between NRF2 signaling and inflammatory response. Inflammation and oxidative stress can cause tissue damage with a complex interplay of processes, indicating that the defensive response to oxidative and electrophilic insults by activation of NRF2 can lead to the synergic anti-inflammatory effects. In addition, many lines of experimental results indicate that the activation of NRF2 signaling modifies inflammatory reaction though multiple pathways (Figure 3) [5].

Nrf2 activation by the triterpenoid (CDDO-Im) induces a series of antioxidant genes, accompanied by suppressed expression of inflammatory-related genes, such as *TNF-* α, *IL-6*, *MCP-1* (monocyte chemo attractant protein-1), and *MIP2* (macrophage inflammatory protein-2) in LPS-stimulated mouse peritoneal macrophages, as well as LPS-treated mice (in which anti-inflammatory effects did not occur in Nrf2-deficient cells and mice) [218]. In mouse peritoneal macrophages stimulated by prostaglandin, Nrf2 induces *Hmox1* and peroxiredoxin 1 (*Prx-1*) gene expression, which appears to inhibit inflammatory-related gene expression [219]. Interestingly, human PRX-1 is reported to negatively regulate macrophage migration inhibitory factor (MIF) [220], a crucial factor in the regulation of inflammation. Accordingly, MIF activity was measured in human urine samples after SFN administration to investigate the possibility for MIF as a biomarker to assess anti-inflammatory efficacy in interventions [142]. SFN seems to affect multiple downstream pathways associated with anti-inflammatory actions and NRF2 signaling may be but one pivotal pathway. Pretreatment of primary peritoneal macrophages by SFN inhibits inflammatory-related gene expression, including *Cox-2* and *iNOS*, which was attenuated in Nrf2-deficient cells [221]. The suppression of *COX-2* expression in PBMCs was indicated in a clinical study, where the treatment response by SFN for autism spectrum disorder patients was examined [195]. The association between Keap1-Nrf2 signaling and the nuclear factor-κB (NF-κB) signaling pathway, one of the essential immune-related pathways, is another potential mechanism underlying anti-inflammatory effects by Nrf2 activation [222,223,224]. Nrf2 and NF-κB individually affect other signaling cascades, in addition to crosstalk between two pathways. The absence of Nrf2 amplifies NF-κB activity, suggesting an inhibitory action of Nrf2 toward NF-κB [225], whereas NF-κB can modulate Nrf2 transcriptional activity [226]. Understanding the molecular links between these two signaling pathways may lead to more informative biomarkers. Intracellular NF-κB signaling molecules have been examined to evaluate responses for inflammatory disease in clinical studies using DMF and BARD-Me [50,66]. Other molecular mechanisms behind Nrf2-mediated anti-inflammatory effects have been described, such as transcriptional suppression of proinflammatory cytokine genes (*Il-6* and *Il-1β*) [227] and the correlation between Nrf2 signaling and NLRP3 inflammasome activity [228].

Leaving aside the linkage of specific molecular pathways indicated above, the general approaches for monitoring anti-inflammatory responses are widely examined as well, using global inflammatory markers such as C-reactive proteins (CRP), cytokines, chemokines, inflammatory lipid mediators, and the immune cell counts.

### 3.5. Carcinogen Metabolites/DNA Adducts

Initial studies probing the functional consequences of enhanced Nrf2 signaling have focused on toxicological models, in which pharmacologic and genetic means were used to alter pathway flux in mice [6,229]. For example, Nrf2-deficient mice had a significantly higher burden of gastric neoplasia after treatment with benzo[a]pyrene than did wild-type mice [113]. Oltipraz significantly reduced the multiplicity of gastric neoplasia in wild-type mice but had no effect on tumor burden in Nrf2-deficient mice. A similar result was observed when SFN was used as the chemopreventive agent in this model [230]. At the same time, biomarkers based on carcinogen metabolites and excreted DNA adducts were being developed and validated as modifiable, short-term endpoints to assess the efficacy of chemopreventive interventions and for cohort selection in clinical trials [231].

**Figure 4:**
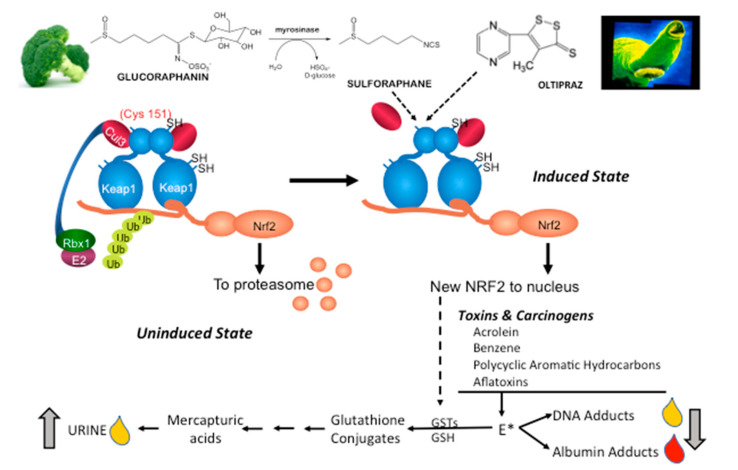
Induction of KEAP1-NRF2 signaling leads to enhanced detoxication of carcinogens in clinical trials. Air-borne (e.g., acrolein, benzene, polycyclic aromatic hydrocarbons) and food-borne (e.g., aflatoxins) carcinogens are metabolized to reactive electrophiles (E*) by cytochrome P450 and other enzymes. NRF2 target genes such as GSTs can conjugate glutathione (GSH) to E*, leading to formation of nonreactive, water-soluble mercapturic acids. E* can also initiate carcinogenesis by forming promutagenic DNA adducts. Some DNA adducts undergo spontaneous or enzymatic depurination allowing for excretion in urine. E* can also form protein adducts with lysine or cysteine residues in albumin. Mercapturic acids and the adducts can be quantified in clinical samples following ambient exposures using mass spectrometric techniques. Cys, cysteine; SH, sulfhydryl; Ub, ubiquitin; Cul3, cullin 3, Rbx1, ring-box 1.

Aflatoxin biomarkers were first used in 1995 as intermediate endpoints in a chemoprevention trial of oltipraz in Qidong, China, a hotspot for the development of liver cancer [100]. Aflatoxin, found in moldy corn, is a potent human hepatocarcinogen. In a placebo-controlled, double-blind study of daily oltipraz, median urinary levels of a detoxication product—aflatoxin-mercapturic acid (a glutathione conjugate derivative)—were elevated six-fold. Increased formation of aflatoxin-mercapturic acid reflects induction of aflatoxin conjugation through the actions of oltipraz on the expression of GSTs and is presumed to be a NRF2-mediated action (Figure 4). When metabolically activated, aflatoxin can also form stable covalent adducts with a lysine residue in serum albumin and the N^7^ atom of guanine in DNA. In as much as albumin has a circulating half-life of about 3-weeks, albumin adducts provide an integrated estimate of sub-chronic to chronic exposures to toxicants. By contrast, the DNA adduct, which rapidly depurinates from DNA and is excreted in urine, reflects exposures within the past 24 h. In the same trial, aflatoxin-albumin adducts were noted to slowly decline during the active intervention phase with oltipraz and to rebound to placebo control levels after cessation of the intervention [99].

A broccoli beverage containing defined concentrations of glucoraphanin was evaluated for its ability to alter the metabolic disposition of aflatoxin [125]. In this study, 200 healthy adults drank beverages containing either 400 or <3 μmol glucoraphanin nightly for 2 weeks. Measurement of urinary levels of SFN metabolites indicated striking inter-individual differences in bioavailability, likely reflecting individual differences in the rates of hydrolysis of glucoraphanin to SFN by the intestinal microflora of the study participants. Accounting for this variability, in a secondary analysis a significant inverse association was observed for the uptake and subsequent urinary excretion of SFN and aflatoxin-N^7^-guanine adducts in individuals receiving broccoli sprout beverage. Changes in the metabolism of phenanthrene-tetraol were also noted in this trial.

Follow-up studies with broccoli-sprout-based interventions in Qidong demonstrated enhanced detoxication of air pollutants over one-week or three-month time frames. Higher urinary excretion levels for benzene and acrolein mercapturic acids were observed [146,154] compared to the placebo beverage. A recent dose-response study with a broccoli sprout beverage indicated that the dynamic range for induction of NRF2-driven detoxication of benzene metabolism to it mercapturic acid was rather limited in participants exposed to ambient air pollutants (~60% elevation) and saturated a relatively low level of SFN dosing [188].

In a study in which smokers were administered phenethyl isothiocyanate (PEITC), the PEITC arm reduced metabolic activation of NNK, one of the most potent lung carcinogens present in cigarettes [232]. Larger increases in rates of excretion of detoxification metabolites (often mercapturic acids) of combustion pollutants such as benzene and aldehydes were observed following PEITC intervention [232]. The mechanisms of action of the two isothiocyanates, SFN and PEITC, are not identical, but do include Nrf2 activation. In a small trial, subjects ingested 250 g each of Brussels sprouts and broccoli per day [233]. At the end of this feeding phase, subjects consumed a cooked meat meal with measured levels of 2-amino-1-methyl-6-phenylimidazo[4,5-b]pyridine (PhIP), a heterocyclic amine carcinogen formed from char-broiling meats) and urine samples were collected. Cruciferous vegetable consumption significantly increased hepatic CYP1A2, as demonstrated by changes in saliva caffeine kinetics. and significantly increased the urinary excretion of N^2^-hydroxy-PhIP-N^2^-glucuronide, another possible detoxication outcome through NRF2 induction in humans.

Many—but certainly not all—environmental carcinogens such as benzene, polycyclic aromatic hydrocarbons, and aflatoxins can be detoxified through the molecular pathways induced by agents such as oltipraz and SFN (Figure 4).

### 3.6. Metabolomics

Widespread application of “omic” technologies is providing precise guidance for selection of therapeutic interventions based on patient biology [234]. Metabolomics is an emerging field of “omics” that characterizes small-molecule metabolites in biological systems. Metabolomic analyses reflect both the steady-state physiological equilibrium of cells or organisms, as well as their dynamic metabolic responses to stimuli, including drugs. The opportunity to use perhaps hundreds of analytes for assessment drug pharmacodynamics, or indeed as descriptors of human health and disease, will provide greater accuracy in unraveling the complexity of human biology. Already, metabolomic profiles obtained prior to, during, or after drug treatment are used to provide insights about the drug mechanism of action and variation of response to treatment [235].

Metabolomics technologies are beginning to be applied to the discovery of biomarkers in clinical studies using NRF2 inducers. Importantly, several untargeted metabolomic studies have been performed [149,183,190]. Bent et al. [182] identified altered urinary metabolites that were correlated with changes in symptoms in patients of autism spectrum disorder by SFN treatment, and which they were clustered into pathways of oxidative stress, amino acid and gut microbiomes, neurotransmitters, hormones, and sphingomyelin metabolism.

Specific metabolite biomarkers are also widely used for monitoring physiological responses and pharmacotherapy in clinical studies. Dyslipidemia is a risk factor for cardiovascular decease and fatty liver. To examine the beneficial effect for dyslipidemia, the lipid profiles in blood samples, such as total cholesterol, triacylglycerol, and lipoproteins (low-density lipoproteins; LDL, high-density lipoproteins; HDL), are investigated in the clinical studies of SFN [122,149,151,161]. It is reported that the activation of Nrf2 represses the expression of key enzymes involved in fatty acid synthesis, with concomitant reduction in the levels of hepatic lipids in mice [236]. Kikuchi et al. indicated that intervention by SFN-rich broccoli sprout extract improved hepatic abnormalities; however, TG, HDL, and LDL-cholesterol were not changed in this study [167]. As these biomarkers are commonly used in clinical diagnostics, they have been allocated as clinical biomarkers in this article (Table S1).

The recent global approach of identifying NRF2 target genes reveals novel gene candidates, including metabolic genes [209,237,238]. In addition to GSH-metabolism-related genes, xenobiotic metabolism, lipid metabolism, glucose metabolism, and several amino acid transporter mediated genes are considered to be NRF2 target genes, which seemingly have expanded the role of NRF2 toward metabolic regulation. Integrated approaches combining metabolomics and genomics could lead to new discoveries of mechanism-based metabolite biomarkers for NRF2 inducers. NRF2 influences heme, iron, and hemoglobin metabolism in human blood cell lines. Furthermore, NRF2 regulates fetal γ-globin gene expression and fetal globin genes [239,240]. Given these findings, NRF2 activation by SFN was introduced to sickle cell disease patients, and mRNA expression of *HbF* and HbF protein levels was examined [172].

## 4. Integrated Assessment of Biomarker Outcomes

**Figure 5:**
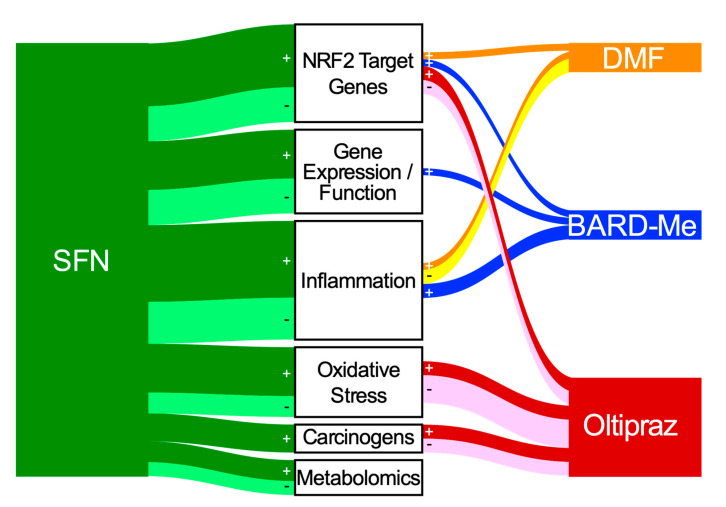
Sankey diagram of mechanism-based biomarkers measured in clinical trials for SFN (green), DMF (orange), BARD-Me (blue), and oltipraz (red). Darker lines (green, orange, blue, red) marked with “+” are studies in which at least 1 biomarker was reported to exhibit a statistically significant (*p* < 0.05) change. Lighter lines marked with “-“ indicate nonsignificant (i.e., null) responses for all biomarkers examined within a study. This accounting overemphasizes positive outcomes. Lines emanating from each inducer are connected to different measured biomarker categories. The box height for each NRF2 inducer and the thickness of the flow lines or nodes emanating from each inducer to the biomarker categories are proportional to the number of biomarker category counts.

Outcomes measured in published clinical studies of NRF2 inducers were identified, listed, and categorized under pharmacokinetics or into six classes of mechanism-based biomarkers: NRF2 target genes, gene expression and function, inflammation, oxidative stress, carcinogen metabolites and adducts, and metabolomics, as well as disease-specific and clinical responses (Table S1). Using data derived from the literature summarized in Table S1, a Sankey plot (Figure 5) was developed to indicate the use and outcomes associated with the mechanistic biomarker measures reported for the four clinically used agents. Measured biomarkers were designated “+” for statistically significant or “-“ for statistically nonsignificant changes, no change, or undetectability in the Sankey diagram. In each clinical study, a biomarker category receives only one count, regardless of the number of specific biomarkers measured in that category. As an example, in the 2009 SFN clinical study by Riedl et al. [134], although several NRF2 target genes (*HMOX1, NQO1, GSTP1, GSTM1)* were measured, the NRF2 target gene biomarker category for SFN still receives only one count for that biomarker category. More detailed presentation of the numbers of individual biomarkers within each classification and their outcomes in the clinical trials is provided in Table 1. Although the Sankey diagram provides a somewhat dire picture of the utility of biomarker measurements for each NRF2 inducer, closer inspection of each specific biomarker presented in Table 1 provides more hopeful insights.

Overwhelmingly, SFN dominated the use of mechanistically based biomarkers in clinical studies, with 77 specific and distinct biomarkers being measured (Table 1); approximately two-thirds (53) exhibited statistically significant responses. Several were modulated significantly in multiple trials. Oltipraz studies collectively utilized only 18 biomarkers, followed by DMF and BARD-Me at 16 and 11 each, respectively.

NRF2 target gene biomarkers were the only biomarker class to be affected positively by all four agents. Nineteen NRF2 target gene biomarkers were measured for SFN, with 14 (74%) biomarker measurements exhibiting statistical significance. Half of the NRF2 biomarkers measured for oltipraz were statistically significant. Unexpectedly, both BARD-Me and DMF only had one biomarker measurement that was statistically significant. Of note, for the most studied NRF2 target gene, *NQO1*, transcript levels showed significant induction in 7 out of 13 settings (54%). Of other transcripts with multiple assessments, only *γGCS, GCLM*, and *GSTP* transcripts showed 50–100% of measurements as statistically significant. However, the majority of the NRF2 target genes measured exhibited changes that were statistically significant in at least one study.

SFN is the primary inducer to manifest statistically significant measurements for gene expression or function biomarkers (8 of 11), although there have been almost no attempts with the other drugs (one positive gene, cyclin D1, in a large set of cancer genes with BARD-Me). Limited assessments of signaling pathways (e.g., TGF-β, EGFR, and insulin) have been undertaken. Modulation of epigenetic marks through inhibition of HDAC activity and subsequent effects of cell proliferation and tumor development may be an important mode of action that is possibly linked to NRF2.

Only SFN and oltipraz trials elicited statistically significant changes of oxidative stress biomarkers. Most oxidative stress biomarkers (9 out of 12 biomarkers) measured in SFN clinical studies were positive; oltipraz studies were less forthcoming (2 of 6). More than half of the oxidative biomarkers exhibiting significant changes utilized either GSH, 8-OHdG, or MDA levels.

Although trials with all four inducers assessed inflammation biomarkers, SFN showed the most positive responses (19), followed by BARD-Me (3), DMF (1), and oltipraz (0). Upon closer inspection of the specific inflammation biomarkers measured, lipid mediators including PGD2, tetranor-PGEM, 11β-PGF2α, and 11-dehydro-TXB2; select chemokines and cytokines; NF-κB pathway markers; and proinflammatory aggregate transcripts appear to be useful biomarkers in these settings.

Multiple clinical trials using either SNF or oltipraz have successfully used the measurements of carcinogen metabolites or adducts to define a pharmacodynamic action. Outcomes dependent on the carcinogens targeted detoxication of air pollutants (benzene, aldehydes) and aflatoxin, which were consistently enhanced, whilst that of benzo[a]pyrene was not.

Metabolomic studies are an emerging approach to developing mechanism-based biomarkers. Within these four agents, only SFN trials have utilized these technologies, albeit with some success (3 out of 5).

## 5. Conclusions

### 5.1. Critical Path for Biomarkers in NRF2 Drug Development?

The clinical development pathways for the four “NRF2 inducers” have been distinct. DMF appears to have been appropriated for treatment of multiple sclerosis in the absence of precise mechanistic understanding of its action in humans beyond the appreciation of its immunomodulatory effects. Biomarkers were of limited use in guiding its regulatory approvals. BARD-Me was selected by Reata Pharmaceuticals as a lead compound from a large series of triterpenoids known to potently activate Nrf2 signaling in cells and animal models. However, biomarker studies in humans have again seemingly played a minor role in its promising clinical development (other than eGFR). Oltipraz was a drug originally developed by Rhône-Poulenc for the chemotherapy of schistosomiasis that was repurposed, largely through the support and guidance of the Chemoprevention Branch of the National Cancer Institute, for evaluation as a cancer chemopreventive agent. Biomarkers were central to these early studies, although issues of drug availability and difficulties related to synthesis (cost) and toxicities short-circuited its development. SFN arose out of extramurally funded academic labs, where small biomarker-driven studies have led to a large series of incremental improvements in formulation, dose selection, and identification of possible cohorts for clinical use. To date, no clinical application shows a clear path to its therapeutic or prophylactic registration. Thus, depending upon the agent, biomarkers have either been critical for their discovery and translational development or ancillary to the process, as can be inferred from the data in Figure 2B.

### 5.2. Metrics of Success and Confounders

As highlighted in Figure 5 and Table 1, a large range of biomarkers have been employed with somewhat limited success in clinical trials involving inducers of the NRF2 pathway. This uneven outcome arises despite many animal studies supporting roles for the pathways and processes that are measured. However, the approach herein of relying on “statistical significance” as the arbiter of a successful outcome oversimplifies the complexity of the challenge. These biomarkers have been studied in different settings with different populations (healthy and diseased), different treatment regimens (dose, schedule, and time of biomarker measurement), and different formulations, even within one agent, as well as differences in the sources of specimens interrogated (PBMCs vs. target cells). Additionally, there is a lack of consensus regarding methodologies and the extent of validation, standardization, and reproducibility of biomarker measurements in these clinical studies. There is also a three-decade time period in across which these trials have been conducted, masking underlying changes in analytic technologies applied to biomarker measures. Finally, it is evident that these compounds can affect NRF2-independent responses and pathways in humans. With this diversity of study populations, analytical methodologies and study designs, and unclear target specificity, it is difficult to reach overarching evaluations of the performance of each biomarker or the degree of association with the NRF2 pathway currently used in the clinical studies. While cognizant of these limitations, there are some comments that can be made regarding both foundational observations and gaps within the clinical studies of the current four agents thought to modulate NRF2 signaling.

### 5.3. Lessons from Dose-Response

Examination of dose-responses of biomarkers provides insights into associations between putative mechanisms of action and clinical outcomes. In prevention studies especially, they can help define minimally effective doses, with disease prevention being a setting in which any side-effects are unacceptable. However, to date there have been very few studies that have examined dose-response in the actions of these agents on NRF2-related biomarker levels in humans. Moreover, some of the studies that attempted such analyses resulted in no modulation of biomarkers at any dose. O’Dwyer et al. [98] did observe increases in *NQO1* and *γGCS* transcripts in PBMCs and colon mucosa with doses of 125 to 250 mg/m^2^ of oltipraz. No additional increases were observed for 500 and 1000 mg/m^2^ doses. Only the lowest doses increased GST and NQO1 activity in PBMCs and colon mucosa; surprisingly, the higher doses were not different from baseline. Riedl et al. [134] reported graded increases in transcript levels of *GSTM, GSTP, HMOX1*, and *NQO1* in nasal lavage samples from participants receiving 3 daily doses of 25 to 200g of a broccoli sprout homogenate to provide SFN. Maximal increases were of the magnitude of 100%. Ushida et al. [241] also reported modest dose-dependent increases in serum GST and NQO1 activity after administering volunteers low doses of 30 or 60 mg of glucoraphanin-based tablets. Lastly, Doss et al. [172] fed participants with 50, 100, or 150 g of a SFN-rich broccoli sprout homogenate and reported significant induction pre- to post-treatment for *HMOX1* mRNA at 150 g, for *HBG1* at 100 g, and no significant change at any dose for *NQO1*. Thus, dose-response data remains elusive to this point, and possibly suggests greater pharmacodynamic action at lower rather than highest doses—a so-called “∩”-shaped curve. In accord with this possibility, Chen et al. [188] reported that the enhanced excretion of benzene mercapturic acids appeared to be saturated at modest doses of a glucoraphanin + SFN-rich beverage. Given that these biomarkers likely represent direct effects on NRF2 signaling, it appears that the dynamic range for pharmacological activation of the pathway might be more limited in humans than in mice.

### 5.4. Take-Home Messages

Despite the variability in the biomarker responses, there are a few overarching conclusions that may be drawn regarding the approaches taken to assess the pharmacodynamic action of NRF2 inducers through the analysis of biomarkers in clinical trials.

- ***NRF2 Target Genes***: Given the preclinical evidence that all 4 of the agents can activate Nrf2 signaling, it is comforting that all four increased activities or transcript levels of classic NRF2 target genes in clinical trials. NQO1 was most studied and showed reasonable consistency across trials. Worryingly, in most studies the induction of *NQO1* transcripts exhibited a limited dynamic range (~ < 2-fold). In an oltipraz study, concordance between expression in surrogate (e.g., PBMCs) and target tissues (e.g., colonic mucosa) was reported. Limited studies suggest possible merit for PCA or clustering analyses to characterize induction “signatures” that may be more revealing than single candidate genes;
- ***Gene Expression/Function*:** Most studies here have focused on pathways affecting cancer development and progression. The most promising outcomes (with SFN) have centered on modulation of epigenetic regulators such as histone deacetylase (HDAC) and histone acetyltransferase activities. One cancer-related gene expression panel was largely unaltered in a BARD-Me study and the other two agents were not evaluated in this context;
- ***Oxidative Stress:*** Many of the workhorse biomarkers of oxidative stress have been applied to clinical studies with SFN and oltipraz, but not the other two drugs. Oxidized DNA products along with DNA strand breaks have shown protective responses in some of the interventions. Studies using the oxidation products of lipids and proteins have been more variable in their responses, although MDA looks promising. The more integrated measures of TAC, TOS, and OSI have not been revealing in limited studies. Cellular GSH levels have been measured frequently and show repeated, albeit still inconsistent, modulation by intervention;
- ***Inflammation:*** In the aggregate over 35 individual inflammation biomarkers have been measured, while barely half evoked a significant response in any study; very few have been evaluated in multiple studies. The context for selection of candidate biomarkers is rarely presented in these studies. The NRF2 target gene *Il-6* [227] shows some responsiveness, while other cytokines such as IL-1, IL-8 IL-13, TNFα, and IFNγ have been null in multiple studies. Lipid mediators including PGD_2_, tetranor-PGEM, 11β-PGF2α, and 11-dehydro-TXB_2_ offer some promise. Subgroup analyses of responders only within a DMF trial exhibited significant reductions in intracellular NF-κB signaling molecules [50];
- ***Carcinogen Metabolism/Adducts:*** Monitoring detoxication metabolites following interventions in study populations provides strong links to canonical NRF2 mechanisms of action. Multiple studies in settings of unavoidable exposures to air pollution and dietary carcinogens highlight successful interventions with oltipraz and SFN. However, such studies require sophisticated mass spectrometry methodologies for metabolite, DNA adduct, and protein adduct quantification. Moreover, interception of all classes of carcinogens and toxins is not achievable. Perhaps phase 0 “microdosing” trials with small, safe amounts of heavy-isotope-labeled substrates can provide an effective means to prioritize tractable exposures [242]. As with all reviewed biomarkers, extrapolation from biomarker change to extent of risk reduction has not been realized;
- ***Metabolomics*:** Targeted and nontargeted metabolomics are beginning to be applied successfully as biomarkers in clinical trials of NRF2 inducers, albeit exclusively to date with SFN. Recent studies in mice have shown the power of these tools to define the impact of modulation of Nrf2 signaling on cancer cell metabolism [243] and the maintenance of health in space flight [244]. Combinations of omics approaches are likely to provide more integrated pictures of the actions of targeted NRF2 activation on early, intermediate, and later events on the pathways of disease prevention and mitigation.

## Figures and Tables

**Table 1:** Significant vs. null (nonsignificant) outcomes of individual biomarker measures.

		DMF		BARD-Me		Oltipraz		SFN		TOTAL		Percent Significant Outcomes
		Sig. Δ	NS	Sig. Δ	NS	Sig. Δ	NS	Sig. Δ	NS	Sig. Δ	NS	
**Nrf2 Target Genes**	Activity											
	NQO1						1	1		1	1	50%
	GST					1	2	1		2	2	50%
	SOD						1			0	1	ALL NULL
	GPX						1			0	1	ALL NULL
	Transcripts											
	*NQO1*	1		1		1	1	4	5	7	6	54%
	*HMOX1*		1					3	6	3	7	30%
	*GCLC*						1		2	0	3	ALL NULL
	*GCLM*							2	2	2	2	50%
	*GSTM*						1	1	2	1	3	25%
	*GSTP*						1	1		1	1	50%
	*UGT*						1			0	1	ALL NULL
	*GPX*						1		1	0	2	ALL NULL
	*γGCS*					2				2	0	100%
	*TR1*							1		1	0	100%
	*LTB4DH*							1		1	0	100%
	*AKR1C1*							1	2	1	2	33%
	*AKR1C2*							1		1	0	100%
	*AKR1C3*								1	0	1	ALL NULL
	*HBG1*								1	0	1	ALL NULL
	*CBR1*							1	1	1	1	50%
	*SLC7A11*								1	0	1	ALL NULL
	PCA cytoprotection/detox/antioxidant							1		1	0	100%
	Nrf2 related genes (aggregated transcripts)											
	*NQO1, HMOX1, AKR1C1, HSP27, HSP70*							1		1	0	100%
**Gene Function/Expression**	HDAC							3	2	3	2	60%
	Histone acetylation							1	1	1	1	50%
	CYP3A4								1	0	1	ALL NULL
	TGFβ pathway							1		1	0	100%
	Epidermal growth factor receptor							1		1	0	100%
	Insulin signaling							1		1	0	100%
	Cancer-related											
	RNA-seq of prostate cancer genes							1		1	0	100%
	p21^WAF/CIP1^								1	0	1	ALL NULL
	Cyclin D1			1						1	0	100%
	STAT3				1					0	1	ALL NULL
	p-STAT3				1					0	1	ALL NULL
	p21				1					0	1	ALL NULL
	Active caspase 3				1					0	1	ALL NULL
	VEGF				1					0	1	ALL NULL
	HIF1α				1					0	1	ALL NULL
	Decorin							1		1	0	100%
	Insulin-like growth factor								1	0	1	ALL NULL
	p16							1		1	0	100%
**Oxidative Stress**	GSH (Glutathione) levels					2	3	2	1	4	4	50%
	8-OHdG and oxidized nucleosides						1	3		3	1	75%
	DNA strand breaks							1		1	0	100%
	PCOOH (phosphatidylcholine hydroperoxide)							1		1	0	100%
	8-isoprostane							1	3	1	3	25%
	TBARS								2	0	2	ALL NULL
	Protein carbonyls								1	0	1	ALL NULL
	TAC (Total antioxidant capacity)							1	2	1	2	33%
	TOS (Total oxidant status)								1	0	1	ALL NULL
	OSI (Oxidative stress index)							1		1	0	100%
	MDA							2		2	0	100%
	Oxidized-LDL							1		1	0	100%
**Inflammation**	Cytokine											
	IL-1		1						2	0	3	ALL NULL
	IL-4								1	0	1	ALL NULL
	IL-6		1					3	3	3	4	43%
	IL-8		1						3	0	4	ALL NULL
	IL-10		1							0	1	ALL NULL
	IL-12		1							0	1	ALL NULL
	IL-13		1						1	0	2	ALL NULL
	IL-17		1							0	1	ALL NULL
	TNFα		1					1	1	1	2	33%
	IFNγ		1						2	0	3	ALL NULL
	Chemokines											
	CCL5								1	0	1	ALL NULL
	MIP-1B (CCL4)		1							0	1	ALL NULL
	MCP-1 (CCL2)		1					2		2	1	67%
	CXCL1		1							0	1	ALL NULL
	IP-10 (CXCL10)							1	1	1	1	50%
	MIG							1		1	0	100%
	Lipid mediators											
	PGD2	1								1	0	100%
	Tetranor-PGEM							1		1	0	100%
	11β-PGF2α							1		1	0	100%
	11-dehydro-TXB2							1		1	0	100%
	NF-kB pathway			1						1	0	100%
	CRP							2	3	2	3	40%
	Immune response											
	WBC counts							1		1	0	100%
	Neutrophil counts								1	0	1	ALL NULL
	Monocyte counts								1	0	1	ALL NULL
	Macrophage counts								1	0	1	ALL NULL
	T cell counts								1	0	1	ALL NULL
	NKT cells								1	0	1	ALL NULL
	CD4+ and CD8+ T-lymphocytes		1	1						1	1	50%
	Proinflammatory genes (aggregated transcripts)							1		1	0	100%
	PCA immune-response genes								1	0	1	ALL NULL
	Others											
	MIF							1		1	0	100%
	SLPI							1		1	0	100%
	CD105+ and iNOS+ cells			1						1	0	100%
	Virus-induced granzyme B production in NK cells							1		1	0	100%
	Serum pepsinogen I and II							1	1	1	1	50%
**Carcinogen Metabolites/Adducts**	Aflatoxin-albumin adducts					1				1	0	100%
	Aflatoxin-DNA adducts							1		1	0	100%
	Aflatoxin mercapturic acid					1				1	0	100%
	Polycyclic aromatic hydrocarbon-DNA adducts						1			0	1	ALL NULL
	Benzo(a)pyrene-7,8-diol-9,10-epoxide adducts						1			0	1	ALL NULL
	Mutagenicity (urine)						1			0	1	ALL NULL
	Acrolein mercapturic acid							2		2	0	100%
	Benzene mercapturic acid							3		3	0	100%
	Crotonaldehyde mercapturic acid							2		2	0	100%
**Metabolomics**	Cystine							1		1	0	100%
	Plasma metabolites							1	1	1	1	50%
	Urinary metabolites							1		1	0	100%
	Metabolites in prostate biopsies								1	0	1	ALL NULL

The abbreviations used are as follows: AKR1C, aldo-keto reductase family 1 member C; CBR, carbonyl reductase; CCL, chemokine ligands; CD, cluster of differentiation; CRP, C-reactive protein; CXCL, C-X-C motif chemokine ligand; CYP3A4, cytochrome P450 3A4; GCLC, glutamate-cysteine ligase catalytic subunit; GCLM, glutamate-cysteine ligase modifier subunit; γGCS, γ-glutamylcysteine synthase, GPX, glutathione peroxidase; GST, glutathione-S-transferase; GSTM, glutathione S-transferase M; GSTP, glutathione S-transferase P; HBG, hemoglobin subunit gamma, HDAC, histone deacetylase; HIF1 α, hypoxia-inducible factor 1α; HSP, heat shock protein; IFNγ, interferon γ; IL – interleukin; iNOS, inducible nitric oxide synthase; IP-10, interferonγ -induced protein 10; LDL, low-density lipoprotein; MCP-1, monocyte chemoattractant protein1; MDA, malondialdehyde; MIF macrophage migration inhibitory factor; MIG, monokine induced by interferon γ; MIP-1β, macrophage inflammatory protein 1β; NFκB, nuclear factor κ β; NKT, natural killer T; NQO1, NAD(P)H: quinone oxidoreductase 1; NS, not significant; p21, cyclin-dependent kinase inhibitor 1; PCA, principal component analysis; PGD2, prostaglandin D2; PGEM, prostaglandin E metabolite; PGF2α, prostaglandin F 2 α; Sig, significant; SLC7A11, solute carrier family 7 member 11; SLPI, secretory leukocyte peptidase inhibitor; SOD, superoxide dismutase; STAT3, signal transducer and activator of transcription 3; TBARS, thiobarbituric acid reactive substances; TGFβ, transforming growth factor β; TNFα, tumor necrosis factor α; TR, thioredoxin reductase; TXB2, thromboxane B2; UGT, UDP-glucuronosyltransferases; VEGF, vascular endothelial growth factor; WBC, white blood cell; 8-OHdG, 8-hydroxy-2′-deoxyguanosine.

## Supplementary Materials

The following are available online at https://www.mdpi.com/2076-3921/9/8/716/s1: Table S1: Summary of literature reporting clinical studies with dimethyl fumarate, bardoxolone methyl, oltipraz, and sulforaphane. Publications including the use of pharmacodynamic biomarkers are highlighted.
